# Motif elucidation in ChIP-seq datasets with a knockout control

**DOI:** 10.1093/bioadv/vbad031

**Published:** 2023-03-16

**Authors:** Danielle Denisko, Coby Viner, Michael M Hoffman

**Affiliations:** Department of Medical Biophysics, University of Toronto, Toronto, ON M5G 1L7, Canada; Princess Margaret Cancer Centre, University Health Network, Toronto, ON M5G 1L7, Canada; Princess Margaret Cancer Centre, University Health Network, Toronto, ON M5G 1L7, Canada; Department of Computer Science, University of Toronto, Toronto, ON M5S 3G4, Canada; Department of Medical Biophysics, University of Toronto, Toronto, ON M5G 1L7, Canada; Princess Margaret Cancer Centre, University Health Network, Toronto, ON M5G 1L7, Canada; Department of Computer Science, University of Toronto, Toronto, ON M5S 3G4, Canada; Vector Institute for Artificial Intelligence, Toronto, ON M5G 1M1, Canada

## Abstract

**Summary:**

Chromatin immunoprecipitation-sequencing is widely used to find transcription factor binding sites, but suffers from various sources of noise. Knocking out the target factor mitigates noise by acting as a negative control. Paired wild-type and knockout (KO) experiments can generate improved motifs but require optimal differential analysis. We introduce peaKO—a computational method to automatically optimize motif analyses with KO controls, which we compare to two other methods. PeaKO often improves elucidation of the target factor and highlights the benefits of KO controls, which far outperform input controls.

**Availability and implementation:**

PeaKO is freely available at https://peako.hoffmanlab.org.

**Contact:**

michael.hoffman@utoronto.ca

## 1 Introduction

Transcription factors, often recognizing specific patterns of DNA sequences called motifs, control gene expression by binding to *cis*-regulatory DNA elements (Mitchell and Tjian, 1989). Accurate identification of transcription factor binding sites remains a challenge (Furey, 2012), with experimental noise further compounding a difficult problem (Kidder *et al.*, 2011). Improving motif models to better capture transcription factor binding affinities across binding sites facilitates downstream analyses on gene-regulatory effects. Higher-quality motifs also promote the exclusion of spurious motifs, obviating costly experimental follow-up.

Chromatin immunoprecipitation-sequencing (ChIP-seq) is a standard approach to locating DNA-binding protein and histone modification occupancy across the genome (Johnson *et al.*, 2007; Robertson *et al.*, 2007). Many steps of the ChIP-seq protocol can introduce noise, masking true biological signal and impeding downstream interpretation (Chen *et al.*, 2012; Head *et al.*, 2014; Kidder *et al.*, 2011; Landt *et al.*, 2012; Park, 2009). Poor antibody quality presents a major source of noise, characterized by low specificity to the target transcription factor or non-specific cross-reactivity. Cross-reactive antibodies often cause spurious pull-down of closely related transcription factor family members. Antibody clonality also contributes to antibody quality. Polyclonal antibodies tend to recognize multiple epitopes, which allows for more flexibility in binding to the desired transcription factor but at the cost of increasing background noise (Kidder *et al.*, 2011).

To address issues of antibody quality, large consortia such as the Encyclopedia of DNA Elements (ENCODE) Project have established guidelines for validating antibodies through rigorous assessment of sensitivity and specificity (ENCODE Project Consortium, 2012; Landt *et al.*, 2012). Other considerable sources of technical noise include increased susceptibility to fragmentation in open chromatin regions (Auerbach *et al.*, 2009) and variations in sequencing efficiency of DNA segments arising from differences in base composition (Kidder *et al.*, 2011). Downstream computational processing further reveals a different type of noise arising from contamination of peaks with zingers, motifs for non-targeted transcription factors (Worsley Hunt and Wasserman, 2014).

Additional control experiments can mitigate the effects of the aforementioned biases. Common types of controls include input and mock immunoprecipitation. Input control experiments isolate cross-linked and fragmented DNA without adding an antibody for pull down. Mock immunoprecipitation control experiments use a non-specific antibody, commonly immunoglobulin G (IgG) (Landt *et al.*, 2012; Head *et al.*, 2014), during the affinity purification step, instead of an antibody to the transcription factor. In theory, IgG mock experiments should better address technical noise since they more closely mimic the steps of the wild-type (WT) ChIP protocol (Landt *et al.*, 2012). In practice, however, they suffer from a range of issues stemming from low yield of precipitated DNA (Kidder *et al.*, 2011). Although the ENCODE Project (ENCODE Project Consortium, 2012) recommends the use of input controls (Landt *et al.*, 2012), experiments using them also suffer from limitations. Input can only capture biases in chromatin fragmentation and sequencing efficiencies, thus failing to capture the full extent of ChIP-seq technical noise.

Knockout (KO) control experiments present an attractive alternative to input and mock immunoprecipitation. In these experiments, mutations directed to the gene encoding the target transcription factor result in little to no expression of the transcription factor, prior to ChIP-seq. This preserves most steps of the ChIP protocol, including antibody affinity purification. Loss of the target transcription factor may result in other changes in cellular gene regulation. This, in turn, may prevent the capture of cell-type-specific artifacts no longer present in the KO cellular state. Such regulatory changes could include altered chromatin accessibility or differences in combinatorial transcription factor binding. KO experiments may also suffer from the pitfalls of mock immunoprecipitation controls, such as low yield. Nevertheless, KO experiments can account for both antibody-related noise and library preparation biases, therefore addressing a large proportion of technical noise in ChIP-seq experiments.

Common transcription factor KO constructs include CRISPR/Cas9-targeted mutations (Cong *et al.*, 2013) and Cre-loxP conditional systems (Schwenk *et al.*, 1995; Sternberg and Hamilton, 1981). In downstream computational analyses, signal from the KO experiment serves as a negative set for subtraction from the WT positive set. Many pre-existing computational methods can use negative sets, typically input controls, to model background distributions (Pepke *et al.*, 2009; Tu and Shao, 2017; Zhang *et al.*, 2014). For example, some peak calling tools, such as MACS2 (Zhang *et al.*, 2008), can perform discriminative peak calling. Most of these tools use the control set to set parameters of a background Poisson or negative binomial distribution (Bailey *et al.*, 2013) serving as a null for assessing the significance of WT peaks (Pepke *et al.*, 2009). A recent comparative assessment (Eder and Grebien, 2022) comprehensively reviews these factors and many others.

Since we expect KO controls to better account for biases in WT data than input controls, optimizing methods for KO controls should generally improve the quality of results from downstream analyses. Indeed, as KO constructs become increasingly more accessible (Doudna and Charpentier, 2014), the need for optimal KO processing guidelines becomes more crucial. While some preliminary studies have investigated the use of KO controls (Krebs *et al.*, 2014; Lun and Smyth, 2016), further rigorous comparison of methods and establishment of a standard remain necessary.

To elucidate motifs when KO controls are available, we introduce a new method, peaKO. PeaKO combines two pipelines incorporating differential processing of WT and KO datasets at different stages. By comparing the rankings of a variety of known and *de novo* motifs, we highlight peaKO’s value for discovering and assessing binding motifs of WT/KO experiments, and peaKO’s applications in other differential contexts.

## 2 Results

### 2.1 PeaKO combines two differential analysis pipelines

**Fig. 1.**
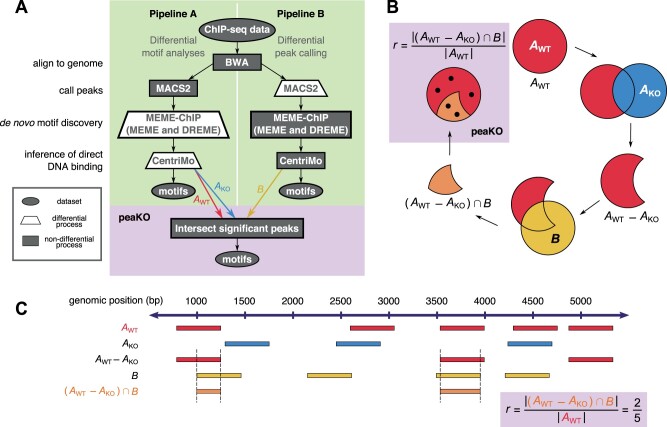
Overview of Pipelines A and B, and peaKO. (**A**) Pipelines A and B differ in their differential analysis steps. Each pipeline accepts both WT and KO ChIP-seq data as input. Pipeline A incorporates differential motif elucidation via MEME-ChIP (Machanick and Bailey, 2011), whereas Pipeline B incorporates differential peak calling via MACS2 (Zhang *et al.*, 2008). Both pipelines produce a ranked list of motifs predicted as relevant to the ChIP-seq experiment by CentriMo (Bailey and Machanick, 2012; Lesluyes *et al.*, 2014). PeaKO extracts significant peaks from CentriMo and computes a new score by which it ranks motifs. (**B**) PeaKO computes its ranking metric *r* through a series of set operations. PeaKO uses peak sets $A_{\text{WT}}$ and $A_{\text{KO}}$, extracted from Pipeline A, and peak set *B*, extracted from Pipeline B. (**C**) A toy example illustrates the calculation of peaKO’s score. Starting from the top row of peak set $A_{\text{WT}}$ and moving downward, we apply the peak set operations of *r* sequentially to identify regions satisfying the numerator criteria

Two steps of ChIP-seq computational processing allow for the subtraction of control signal from WT signal: peak calling and motif analysis. Therefore, we created two complementary pipelines, Pipeline A and Pipeline B, integrating the same software tools but selecting opposing steps to subtract matched KO signal from WT signal (Fig. 1A).

Pipeline A incorporates differential motif analysis through MEME-ChIP (Machanick and Bailey, 2011; Ma *et al.*, 2014). It focuses on the motif discovery algorithms MEME (Bailey and Elkan, 1994, 1995) and DREME (Bailey, 2011), and includes the motif enrichment algorithm CentriMo (Bailey and Machanick, 2012; Lesluyes *et al.*, 2014). MEME-ChIP uses control peak sets for discriminative enrichment analysis (Ma *et al.*, 2014).

Instead of differential motif analyses, Pipeline B incorporates differential peak calling through MACS2 (Zhang *et al.*, 2008). MACS2 uses the control peak set to set the parameters of the background null distribution from which it calls significant peaks. Pipeline B drew inspiration from the KO implemented normalization (KOIN) pipeline (Krebs *et al.*, 2014).

Both pipelines conclude by executing CentriMo (Bailey and Machanick, 2012; Lesluyes *et al.*, 2014). CentriMo’s measure of motif central enrichment assesses the direct DNA binding of the enriched transcription factor (Bailey and Machanick, 2012). Some aspects of CentriMo’s output differ according to whether we choose differential (Lesluyes *et al.*, 2014) or non-differential (Bailey and Machanick, 2012) mode. Both pipelines, however, output a list of motifs ranked in order of increasing p-values. Ideally, the top motif should reflect the target factor in the underlying ChIP-seq experiment, although some circumstances may preclude this.

Each pipeline incorporates a unique approach to discriminative analysis. By modeling the peak background distribution using the negative control set, Pipeline B directly compares the position of read pileups between positive and negative datasets. In this model, we assume that read pileups shared between both datasets represent technical noise, while the remaining significant WT read pileups represent binding of the target transcription factor. Conversely, Pipeline A disregards the positional information of peaks and instead focuses on the position of the motif matches within the peaks. Pipeline A takes into account each peak’s membership in the positive or negative set only when assessing the statistical significance of a motif. In Pipeline A, the simple motif discovery tool DREME compares the fraction of *de novo* motif matches in WT sequences to KO sequences. We assume that motifs more often located near peak centers in the WT dataset than in the KO dataset suggest associated binding events. To preserve signal that may arise from weakly bound sites and contribute to intricacies in binding patterns (Rastogi *et al.*, 2018), we apply no filtering or subsetting of peak sets output from either pipeline. This also allows the application of additional filtering approaches downstream.

To select for motifs that both have consistent matches within peaks and fall within regions of significant read pileup, we combined both pipelines in a new way to develop peaKO. For each motif, peaKO computes the number of overlapping peaks between peak sets generated by both pipelines, with overlaps interpreted as genuine binding events (Fig. 1B–C; see Methods).

### 2.2 PeaKO can improve or maintain rankings of the known motif over either differential pipeline

To assess the performance of each method, we can first compare how well methods rank known canonical motifs of sequence-specific transcription factor datasets. We collected publicly available WT/KO paired ChIP-seq datasets for eight sequence-specific transcription factors (Table 1): ATF3 (Zhao *et al.*, 2016), ATF4 (Han *et al.*, 2013), CHOP (Han *et al.*, 2013), GATA3 (Wei *et al.*, 2011), MEF2D (Andzelm *et al.*, 2015), OCT4 (King and Klose, 2017), SRF (Sullivan *et al.*, 2011) and TEAD4 (Joshi *et al.*, 2017). We evaluated our methods on these datasets, supplementing CentriMo with the collection of vertebrate motifs from the JASPAR 2016 database (Mathelier *et al.*, 2016) (see Methods). Each transcription factor in our WT/KO datasets contains a corresponding motif within the JASPAR database. We used these JASPAR motifs as our gold-standard known motifs, and compared their rankings across methods. As a control, we processed the WT dataset alone through the same pipeline steps without any KO data.

**Table 1.** ChIP-seq datasets used, with associated GEO accession numbers (where applicable) and number of replicates

Factor	GEO	Reference	WT	KO	Input
ATF3	GSE74355	Zhao *et al.* (2016)	1	1	0
ATF4	GSE35681	Han *et al.* (2013)	3	3	0
CHOP	GSE35681	Han *et al.* (2013)	1	1	0
GATA3	GSE20898	Wei *et al.* (2011)	1	1	0
MEF2D	GSE61391	Andzelm *et al.* (2015)	3	1	3
OCT4	GSE87822	King and Klose (2017)	3	3	1
SRF	–	Sullivan *et al.* (2011)	1	1	0^a^
TEAD4	GSE82190	Joshi *et al.* (2017)	1	1	1

a We excluded the available dataset because it came from a different, older DNA sequencer and lacked quality scores.

**Fig. 2.**
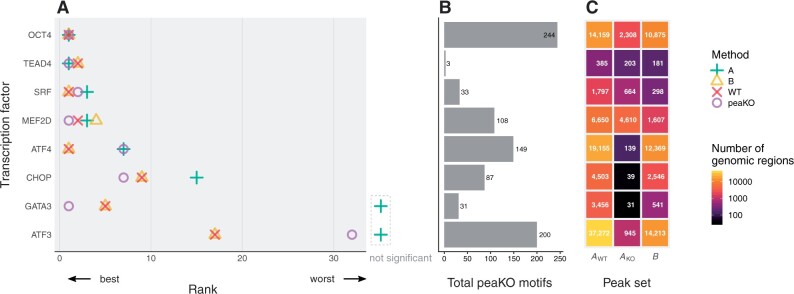
Known transcription factor motifs elucidated by different methods. Motifs originated from the JASPAR 2016 motif database (Mathelier *et al.*, 2016). KO datasets served as a control for differential analyses. (**A**) Each method ranked JASPAR database motifs based on their centrality within peak sets, as determined by CentriMo (Bailey and Machanick, 2012; Lesluyes *et al.*, 2014). Ranks correspond to the ChIPped transcription factor’s known motif (Table 2). Dashed area to right of plot: motifs without statistical significance. (**B**) Total number of motifs assessed by peaKO. (**C**) The number of peaks found by each method varies across peak sets

In five out of eight cases, peaKO improved or maintained the optimal rank relative to all other methods. PeaKO also always improved or maintained the rank relative to at least one other method (Fig. 2). The total number of ranked motifs differed between experiments, which suggests that peaKO may benefit analyses for a wide range of transcription factors with variable binding affinities. Of the other methods, Pipeline A performed the worst overall, as exemplified by non-significant Fisher E-values for both the GATA3 and ATF3 datasets. Pipeline B performed similarly to the use of only WT data processed without controls. Both of these procedures achieved or maintained the best rank in four cases. This suggests that Pipeline B benefits little from the control. PeaKO combines the best aspects of both types of differential analysis pipelines, limiting their deficiencies and highlighting their strengths. This generally leads to better rankings of known motifs.

### 2.3 *De novo* motifs consistently match known motifs

We investigated each method’s ability to rank *de novo* motifs and assessed the similarity between *de novo* and known JASPAR motifs. For consistency, we pooled *de novo* motifs generated by each method (see Methods). We quantified similarity between *de novo* and known motifs using Tomtom (Gupta *et al.*, 2007). We studied these methods on the same eight WT/KO paired datasets used for our known motif analyses.

**Fig. 3.**
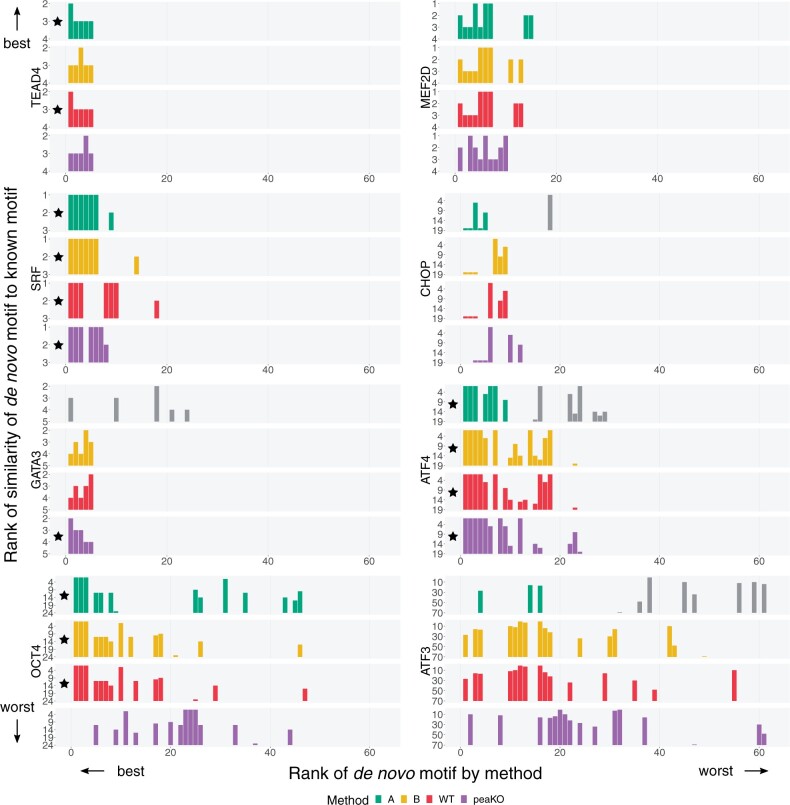
Similarity of discovered *de novo* motifs to canonical JASPAR motifs across four methods. For eight transcription factors (Table 2), we ran four methods (green: Pipeline A, yellow: Pipeline B, red: WT alone and purple: peaKO) on a pooled set of *de novo* motifs generated by MEME (Bailey and Elkan, 1994, 1995) and DREME (Bailey, 2011). Each method generated a ranking of *de novo* motifs (*x*-axis). For each of these motifs, we quantified similarity to the known motif using Tomtom (Gupta *et al.*, 2007) (*y*-axis). For a given *de novo* motif, Tomtom finds and ranks the most similar motifs from the total set of JASPAR motifs. We record the rank of the transcription factor’s known JASPAR motif within this list. To emphasize strong matches to known motifs, the provided ranks lie in descending order, with the best (rank 1) motif, at the top. In some cases, the best rank achieved by the match does not reach 1, as reflected by a greater lower limit. Black stars: methods achieving the best possible rank (of 1, for method rankings and the lower limit rank, for similarity rankings) across both ranking schemes within each experiment. Gray bars: motifs that were not statistically significant and gave rise to arbitrary rankings

Usually, top *de novo* motifs more closely resembled the canonical motif across methods, resulting in most ranking near 1 (Fig. 3). Conversely, motifs ranking lower tended to have fewer matches to the known motif, often not even matching the known motif at all. PeaKO generally followed this trend, but in a few exceptions, such as CHOP, OCT4, and ATF3, top motifs also sparsely matched the canonical motif. PeaKO might have found related, interacting factors, rather than the factor of interest. For example, many top *de novo* motifs reported by peaKO for the CHOP dataset closely matched the motif for ATF4, which interacts with CHOP (Han *et al.*, 2013).

### 2.4 PeaKO can differentiate similar GATA family motifs within the same ChIP-seq experiment

We delved deeper into our GATA3 results, for which peaKO outperformed all other methods. GATA3 belongs to the family of GATA factors, all of which bind GATA-containing sequences (Merika and Orkin, 1993). Despite having similar motifs, each GATA factor plays a distinct role and usually does not interact with the others (Viger *et al.*, 2008).

Distinguishing the targeted motif among GATA factors and other large transcription factor families often presents a challenge. Minor differences in position weight matrices (PWMs) (Berg and von Hippel, 1987) can cause major differences in genome-wide transcription factor binding sites (Lai *et al.*, 1993). Understanding the downstream effects of transcription factor binding necessitates pulling apart these intricacies in motif preferences.

**Fig. 4.**
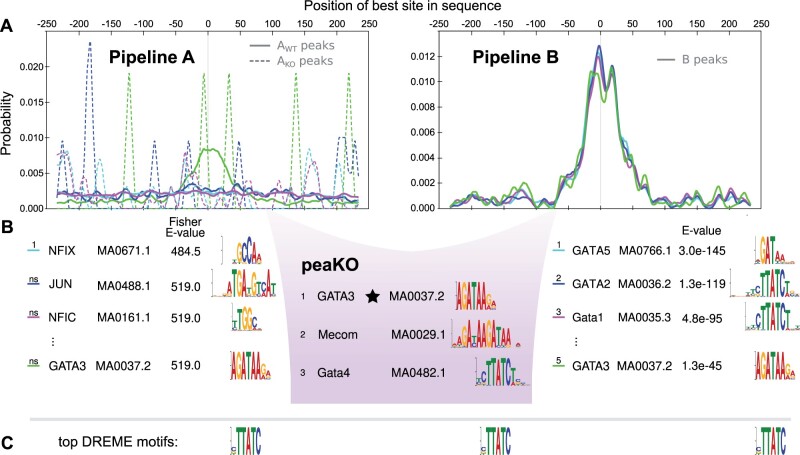
PeaKO ranks the GATA3 motif above other GATA transcription factor family motifs. (**A**) CentriMo (Bailey and Machanick, 2012; Lesluyes *et al.*, 2014) probability plots depict enrichment of the top three motifs from each method, along with the GATA3 motif, within peak sets. (**B**) Motifs resulting from each pipeline and peaKO lie beneath associated CentriMo plots. Motifs and corresponding sequence logos (Schneider and Stephens, 1990) originate from JASPAR 2016 (Mathelier *et al.*, 2016). Capitalization is as it occurs in JASPAR. The information content of bases in the sequence logos ranges from 0 to 2 bits. Ranks of ‘ns’ indicate non-significant motifs, and therefore unreliable rankings. The black star denotes achieving the best rank of the GATA3 motif. (**C**) Top DREME (Bailey, 2011) motifs with length greater than 5 bp, for comparison. In this case, all three motifs are identical

CentriMo results across both pipelines further reinforced the difficulty of distinguishing these motifs (Fig. 4). Pipeline B identified closely related GATA family members with ranks 1–4, above the desired fifth-ranked GATA3 motif. Pipeline A proved less promising, failing to reliably rank any GATA family members. Furthermore, none of the shown Pipeline A motifs appeared centrally enriched within WT peaks. Instead, we observed a uniform distribution among the WT peak set and a series of stochastic, sharp peaks among the KO peak set, likely representing inflated probabilities due to low sample size.

Despite the difficulties affecting Pipeline B, peaKO draws on its ability to detect GATA family members, and surpasses it by ranking GATA3 first. In this case, peaKO achieves specificity in ranking motifs in the presence of many similar familial motifs.

### 2.5 Low-quality datasets pointing to off-target binding may account for poor rankings across methods

**Fig. 5.**
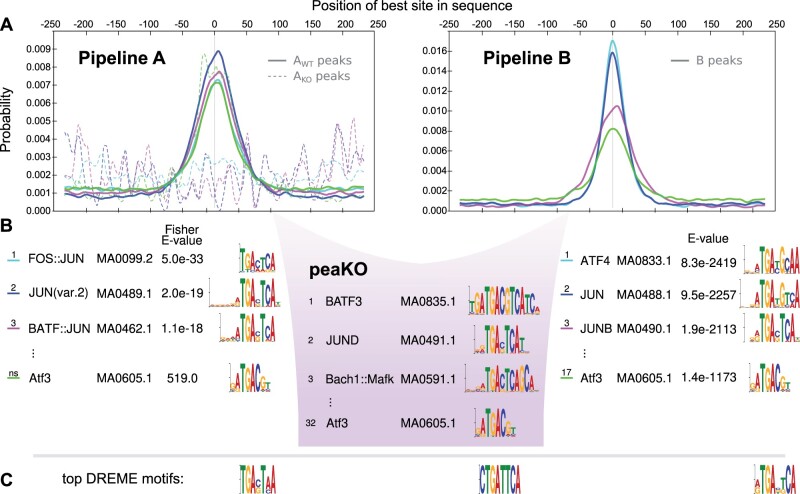
The ATF3 motif is centrally enriched in the ATF3 KO dataset. (**A**) CentriMo (Bailey and Machanick, 2012; Lesluyes *et al.*, 2014) probability plots depict enrichment of the top three motifs from each method, along with the ATF3 motif, within peak sets. (**B**) Motifs resulting from each pipeline and peaKO lie beneath associated CentriMo plots. Motifs and corresponding sequence logos (Schneider and Stephens, 1990) originate from JASPAR 2016 (Mathelier *et al.*, 2016). Capitalization is as it occurs in JASPAR. Information content of bases underlying motifs range from 0 to 2 bits. Ranks of ‘ns’ indicate non-significant motifs, and therefore unreliable rankings. (**C**) Top DREME (Bailey, 2011) motifs with length greater than 5 bp, for comparison

In a few cases, peaKO performed worse than the other methods at ranking the canonical motif (Fig. 2). In particular, we observed a large spread in rankings across methods for ATF3 (ranging from rank 17 to non-significant ranks, above 80). We found central enrichment of the canonical ATF3 motif in the KO peak set, as depicted by Pipeline A’s CentriMo results (Fig. 5). This central enrichment appears even more prominent than that in the WT peak set.

Although CentriMo probabilities depend on the total number of peaks in each set, and a relatively low number of peaks in the control set can inflate these probabilities, we expect non-specific matches to generate a uniform background distribution rather than a distinctive centrally enriched pattern (Bailey and Machanick, 2012; Lesluyes *et al.*, 2014). Accordingly, ATF3 enrichment deviates substantially from our expectations and suggests issues with the underlying KO ChIP experiment. While we cannot exclude other possibilities, this may explain the poor rankings of ATF3 across methods, including peaKO.

### 2.6 KO-controlled analyses improve motif elucidation over input controls

To investigate whether KO controls would better approximate WT ChIP-seq experimental noise than input controls, we used input controls to repeat our analyses. We ran our methods on MEF2D, OCT4, and TEAD4 datasets, which contained input controls (Table 1), by applying the same procedures but using only the input dataset for differential analysis steps.

**Fig. 6.**
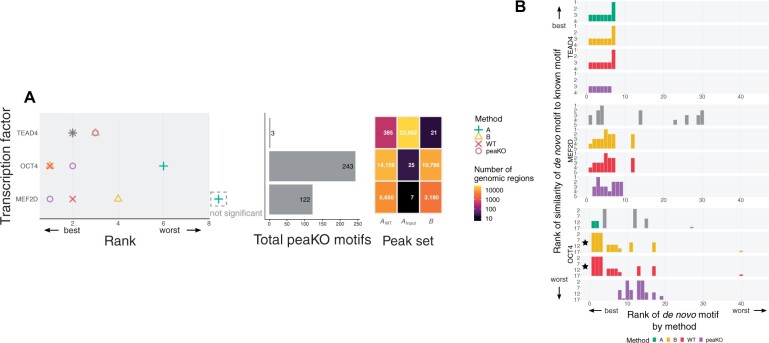
Ranks of known and discovered motifs using input controls. (**A**) Ranks of known JASPAR (Mathelier *et al.*, 2016) motifs across methods for each ChIP-seq experiment (Table 2). Input datasets served as a control in differential analysis steps. Dashed area to right of plot: motif without statistical significance. (**B**) We plotted ranks of *de novo* motifs discovered by MEME (Bailey and Elkan, 1994, 1995) and DREME (Bailey, 2011) against their similarity to the known JASPAR motif, as quantified by Tomtom (Gupta *et al.*, 2007). We compared queried motifs against the JASPAR 2016 target motif database. Gray bars: motifs that were not statistically significant and gave rise to arbitrary rankings. Black stars: methods achieving the best possible rank (rank of 1 for method rankings and lower limit rank for similarity rankings) across both ranking schemes within each experiment

**Fig. 7.**
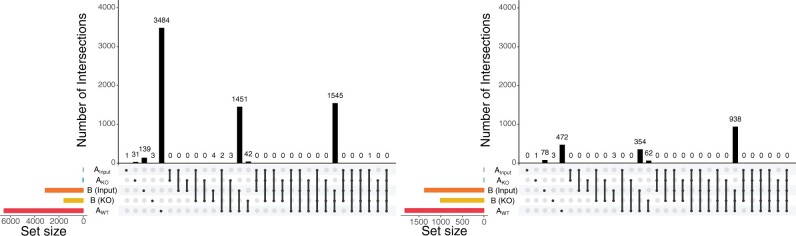
Comparison of peak sets. UpSet plots (Lex *et al.*, 2014) of overlap between MEF2D peak sets, generated by Intervene (Khan and Mathelier, 2017): (*left*) for all motifs and (*right*) for the MEF2D motif only. For Pipeline B peak sets, parentheses indicate the type of negative control used for peak calling: input or KO

Using an input control instead of a KO control usually worsened the ranking of the known motif, as observed by an overall shift across methods toward poorer rankings (Fig. 6A). In *de novo* motif analyses with input controls, top-ranked motifs tended to have slightly poorer matches to known motifs across methods when compared with KO controls (Fig. 6B). As in WT/KO analyses of OCT4, we observed sparsity in top-ranked peaKO motifs matching the known motif. This could point to low affinity of the antibody to the target factor or other types of noise affecting primarily the WT set. Indeed, input experiments yielded even fewer significant peaks from CentriMo than KO experiments (Fig. 7).

Overall, using input controls instead of KO controls led to poorer rankings across methods. Although peaKO did not outperform the other methods using only input, it generally performed similarly, suggesting utility in other differential applications. While this suggests that our method remains useful in this context, integrating input and KO controls may allow future differential methods to perform even better.

## 3 Discussion

Increased accessibility of KO experiments presents a need for standardized computational processing workflows. With KO data, peaKO’s dual pipeline approach generally outperformed each pipeline alone when ranking the known motifs. This holds true even in challenging cases, such as distinguishing among large transcription factor families with shared core motifs. Applying our methods to datasets containing both input and KO controls demonstrates the superiority of KO controls for motif elucidation.

We observed a common theme throughout our analyses pertaining to the characteristic performance of each pipeline alone. When tasked with ranking the known motif, Pipeline A generally produced inferior rankings, especially for ATF3 and GATA3 (non-significant ranks of canonical motifs) and, to a lesser extent, CHOP (rank 15). We could only attribute this to poor experimental quality for ATF3. The significance of differential mode CentriMo p-values, calculated using Fisher’s exact test, appears closely linked to the relative size of each peak set. Both CHOP and GATA3 KO control sets had fewer than 50 KO peaks (Fig. 2), which might account for Pipeline A’s poor performance.

Pipeline B suffered from a different issue: it ranked known motifs almost identically to WT processing alone, without any controls. In some cases, WT processing alone even surpassed Pipeline B. WT-only processing out-performed Pipeline B when using KO and input controls for MEF2D and when using input controls for TEAD4. Since the sole difference between Pipeline B and WT-only processing lies in the peak calling step, identical rankings indicate the sufficiency of constructing the background distribution with WT-derived values alone. Indeed, similar rankings may point to a shortcoming in comprehensively modeling noise captured in KO and input controls. Future work should investigate the robustness of peak callers in modeling control signals and explore potential integration of other tools designed for identifying differential peak sets. For example, DiffBind (Stark and Brown, 2011), built on edgeR (Robinson *et al.*, 2010) and DESeq2 (Love *et al.*, 2014) RNA-seq packages, can find differentially bound sites from sets of peaks, as can ChIPComp (Chen *et al.*, 2015). Differential peak calling with KO controls does, however, reduce the size of the WT peak set.

Perhaps Pipeline B differential peak calling improves an already specific peak set such that the improvement is largely undetectable when ranking known motifs. Nonetheless, rankings differ in some cases and *de novo* motif analyses reveal differences between Pipeline B and WT-only processing. Overall, both pipelines show strengths in specific contexts, which peaKO emphasizes.

We chose MACS2 (Zhang *et al.*, 2008) for peak calling due to its widespread use in ChIP-seq processing and its integration into the ENCODE uniform ChIP-seq processing pipeline (Kundaje *et al.*, 2018). Future research could examine how the size of each peak set impacts downstream steps by comparing different peak recovery strategies.

In selecting known canonical motifs as ground truths to assess our experiments, we limited our ability to evaluate each method’s detection of higher quality motifs. We partially addressed this limitation by finding *de novo* motifs in a discovery use case. Maintaining consistency in performance evaluation across analyses, however, required comparisons of *de novo* motifs to their cognate JASPAR motifs. Therefore, even in the discovery use case, we remain limited by the quality of the known canonical motifs.

Some of our methods ranked the motif of interest less favorably than other GATA family member motifs. GATA family members share a common core motif, yet each have distinct and detectable binding preferences that contribute to their diversity in genome-wide occupancy and function (Merika and Orkin, 1993). Finding the general familial motif could prove sufficient in some cases (Sandelin and Wasserman, 2004). Nonetheless, finding the specific motif helps with understanding the roles of individual transcription factors.

The GATA3 motif (MA0037.2) (Mathelier *et al.*, 2016) that we ranked first (Fig. 4) was generated from 4628 curated sites from a ChIP-seq experiment. This motif likely has more similarity to actual GATA3 binding sites than the other GATA family motifs we compared against, in that it was elucidated via an antibody attempting to specifically target GATA3. CentriMo selected this particular motif as the most enriched match, choosing it over other GATA3 motifs in JASPAR. Even if this motif does not model the full intricacies of GATA3 binding, one would still prefer it ranked above motifs from assays targeting other GATA family members. Our ability to rank the target motif first mainly provides additional confirmation that peaKO performs well and likely has utility in other contexts, including those focused on differentiating similar motifs.

For OCT4 (also known as POU5F1), we selected the Pou5f1::Sox2 motif (MA0142.1). SOX2, like OCT4, regulates pluripotency in embryonic stem cells (Zeineddine *et al.*, 2014). The two transcription factors often act together to regulate gene expression by forming a complex and co-binding to DNA (Aksoy *et al.*, 2013). Here, however, the heterodimer motif differs substantially from the OCT4 motif alone, as it additionally contains a SOX2 motif (Aksoy *et al.*, 2013). We chose to use the heterodimer motif in assessing our methods because the authors of the study that generated the OCT4 dataset found a substantially larger proportion of peaks containing the heterodimer (44.0%) when compared with the monomer (20.6%) (King and Klose, 2017). Upon re-running our analyses using the monomer motif instead, we found poorer rankings across methods, as expected from this imbalance of motif types in peaks (see https://doi.org/10.5281/zenodo.3338330). Higher occupancy of the heterodimer form, however, does not preclude the transcription factor from binding DNA in its monomer form. Although all methods found the heterodimer motif as the top rank, deciding upon which motif form to use and how it affects downstream processing would benefit from further exploration.

We highlighted the ATF3 experiment as a case where peaKO performs poorly, and attributed the poor performance largely to the dataset’s poor experimental quality. Nonetheless, the low information content of the ATF3 motif used as ground truth may supply an alternative explanation. In the JASPAR 2016 motif database, ATF3 motif MA0605.1 captures a single core TGAC motif (Mathelier *et al.*, 2016), derived from *in vitro* protein-binding microarray experiments. A newer version of this motif [MA0605.2, added in JASPAR 2020 (Fornes *et al.*, 2020)], however, contains the canonical ATF/CRE motif, which appears to better represent ATF3’s typical *in vivo* homo- or heterodimerization (Rodríguez-Martínez, 2017). The top motifs returned by peaKO and other methods include many bZIP factor motifs that appear similar to this newer version. Indeed, some of these top motifs themselves derive from *in vivo* experiments. This suggests that peaKO may have performed better than we were able to assess with JASPAR 2016. This ranking also highlights a key limitation to our methods’ assessment.

Further work should incorporate information about transcription factor paralogs and the context of motif derivation: *in vitro* or *in vivo*. This may contribute a more comprehensive reflection of each method not captured by our current framework.

Only a limited amount of KO transcription factor data exists. In this work, we aim to provide convenient and viable methods to process these datasets and further motivate their generation. Even if our methods do not represent some optimal differential peak calling method, we provide a means of rapidly assessing and comparing KO datasets. Similar to KOIN (Krebs *et al.*, 2014), we further extend prior work in this area, facilitating additional iterative comparisons and improvements. As more KO ChIP-seq data of this type materializes, we anticipate that more informative comparisons and analyses will become feasible.

Our use of cross-species PWMs potentially limits our findings. We used motifs from the JASPAR vertebrate collection interchangeably where the known motif did not always originate from the same species as our ChIP-seq datasets (see Tables 1 and 2). Recently, Lambert *et al.* (2019) found that, contrary to commonly held belief, extensive motif diversification among orthologous transcription factors occurs quickly as species diverge. Additionally, PWMs (Berg and von Hippel, 1987) themselves, while providing the most commonly used motif model (Benos *et al.*, 2002; Kulakovskiy *et al.*, 2013a), may not sufficiently capture nuanced binding differences (Dror *et al.*, 2016; Kulakovskiy *et al.*, 2013a).

**Table 2.** JASPAR CORE 2016 (Mathelier *et al.*, 2016) vertebrate motifs used in this study

Common name	JASPAR ID	Motif name^a^
ATF3	MA0605.1	Atf3
ATF4	MA0833.1	ATF4
CHOP	MA0019.1	Ddit3::Cebpa
GATA3	MA0037.2	GATA3
MEF2D	MA0773.1	MEF2D
OCT4	MA0142.1	Pou5f1::Sox2
SRF	MA0083.3	SRF
TEAD4	MA0809.1	TEAD4

a Sentence case motif names designate mouse transcription factor motifs, while full upper case names designate human motifs. Double colons designate heterodimer motifs.

Technical biases can negatively influence peaKO’s scoring metric. Antibody quality, sequencing biases, and various batch effects can lead to a failure to recover sufficient peaks or can enrich for additional off-target peaks. This can, in turn, alter the proportion of peaks in peaKO’s score, changing the motif ranking, in a potentially confounded manner. Further work is needed to assess the statistical robustness of peaKO’s score in the face of such biases. Even without that assessment, our empirical results demonstrate that peaKO’s score remains useful. Furthermore, as we have previously discussed, ranking discrepancies between motifs become obvious in peaKO results. PeaKO, like almost all software that works to elucidate transcription factor binding sites, requires sequence-specific transcription factors that are suitable for narrow peak calling. While this includes the majority of transcription factors, it implies that this method is not applicable for the analysis of histone marks or other broad peak targets. Irrespective of improved motif rankings, peaKO facilitates differential motif comparisons and the generation of potentially improved *de novo* motifs.

Recently, Eder and Grebien (2022) undertook a comprehensive assessment of peak callers, focused in a differential context. While that work addresses a similar problem to that presented here, it differs in several key respects. We have specifically tuned our methods for use with KO datasets, whereas Eder and Grebien’s (2022) work has a less specialized scope. Additionally, they assessed methods using simulated reads. While helpful for their broader focus, such an analysis proves less suitable for our work: we specifically calibrated and tested our method to ensure its robustness against complex, challenging-to-simulate biases present in real datasets.

Overall, Eder and Grebien (2022) find that selecting a peak caller depends highly on scenario, but they support our use of MACS2. They report that after accounting for all factors, MACS2 (Zhang *et al.*, 2008) generally outperforms other callers and DiffBind (Stark and Brown, 2011) only outperforms it slightly in limited scenarios. Our own modifications to the differential peak calling workflow introduce an orthogonal contribution that would noticeably impact this analysis for real data. Accordingly, Eder and Grebien (2022) serves to bolster our methodological choices, and suggests that one could make only small gains by applying different peak calling algorithms. By comparing with a KO control, our analysis provides a high-quality example of the closest to biological ground-truth that we have at the moment. Indeed, one could apply our method to iteratively design more realistic simulated reads for improved comprehensive analyses. In this sense, our work is complementary to existing analyses, and case-study comparisons would prove less useful at this stage, given the limited amounts of KO data.

Lastly, we used peaKO along with our other methods to assess the benefit of KO controls over input, suggesting that peaKO may prove useful for other non-WT/KO differential contexts. Like input, mock immunoprecipitation controls such as IgG control experiments capture background and other non-specific binding signal. Background signal, however, does not directly correspond to the true negative signal captured in KO experiments. Thus, under input and IgG control contexts, all methods can erroneously recover signal arising from antibody cross-reactivity, thereby biasing the motif analyses. CRISPR epitope tagging ChIP-seq (CETCh-seq), which involves the insertion and expression of FLAG epitope tags on the target transcription factor (Savic *et al.*, 2015), presents an alternative differential context which may gain from peaKO. CETCh-seq provides a substantial advantage over traditional ChIP-seq because it only requires one high-quality monoclonal antibody recognizing the FLAG antigen across any number of transcription factor experiments. Notably, CETCh-seq also avoids artifacts that may arise from perturbations of the cellular context in traditional KO ChIP-seq experiments. In particular, CETCh-seq likely preserves downstream regulatory changes conferred by the presence of the factor of interest. Preliminary analyses using CETCh-seq datasets revealed challenges arising from unexpected signal from a shared control of ChIP-seq in an untagged cell line. Further work should investigate the role of CETCh-seq controls and how they integrate with peaKO. We expect this work to also prove useful for comparable or enhanced methods, like cleavage under targets and release using nuclease (CUT&RUN) (Skene *et al.*, 2018), which one could similarly improve through careful design of complementary controls and differential motif analyses.

Similar considerations for the proper use of control sets could also apply to combining replicates. Combining negative control replicates with the irreproducible discovery rate (IDR) framework (Li *et al.*, 2011) may pose problems considering that these datasets represent noise rather than a full range across true signal and noise. This may present an issue as IDR’s underlying copula mixture model assumes the existence of an inflection point within the dataset marking the transition between true signal and noise (Li *et al.*, 2011).

## 4 Conclusion

We present peaKO, a free and publicly available tool for ChIP-seq motif analyses with KO controls (https://peako.hoffmanlab.org). PeaKO improves over two kinds of differential processing in ranking the motif of interest. We anticipate that peaKO will prove useful in identifying motifs of novel transcription factors with available KO controls. We hope this will encourage both greater collection and wider usage of KO datasets.

## 5 Methods

### 5.1 Overview of ChIP-seq processing and analysis methods

ChIP-seq processing follows this overarching path:

1. subject sequenced reads to trimming and quality control assessment;
2. align reads to a reference genome;
3. call peaks according to significant read pileups and
4. elucidate *de novo* motifs and assess peaks for evidence of direct DNA binding.

For some methods, steps 3 and 4 can incorporate information from control datasets. We constructed two pipelines to compare differential analyses in both of these steps (Fig. 1A).

In Pipeline A, we perform differential analysis with MEME-ChIP (Machanick and Bailey, 2011; Ma *et al.*, 2014). MEME-ChIP uses the *de novo* motif elucidation tools MEME (Bailey and Elkan, 1994, 1995) and DREME (Bailey, 2011), and assesses the central enrichment of motifs in peaks via CentriMo (Bailey and Machanick, 2012; Lesluyes *et al.*, 2014). CentriMo ranks motifs according to multiple-testing corrected binomial p-values in non-differential mode (Bailey and Machanick, 2012) or Fisher’s exact test p-values in differential mode (Lesluyes *et al.*, 2014).

In Pipeline B, we perform differential peak calling through MACS2 (Zhang *et al.*, 2008). While Pipeline B draws inspiration from the KOIN pipeline (Krebs *et al.*, 2014), it does not incorporate the HOMER makeTagDirectory or annotatePeaks (Heinz *et al.*, 2010) steps. We replaced HOMER motif tools (Heinz *et al.*, 2010) with those from the MEME Suite (Bailey *et al.*, 2009, 2015). Both Pipelines A and B incorporate identical pre-processing and alignment steps, described later. Since both pipelines employ CentriMo in their last step, they generate a list of ranked motifs with predicted association to the ChIP-seq experiment.

### 5.2 PeaKO: motivation and score

Differential peak calling and differential motif analysis address the same problem of noise removal, albeit in distinct ways. Therefore, we surmised that by combining the two approaches, the results from each pipeline could complement and strengthen one another. CentriMo produces a ranked list of motifs, and each motif has an associated peak set containing a centered window enriched for that motif. We reasoned that motifs with a large proportion of peaks shared between both pipelines are likely relevant to the ChIP-seq experiment. We then created a metric that captures this.

PeaKO takes as input the CentriMo output of each pipeline. We modified CentriMo code to output negative control set peaks associated with each motif in differential mode, since at the time of this work, the software only output positive peaks. These changes have now been incorporated into the MEME Suite and are available from all versions since 5.0.0. From the CentriMo results, peaKO filters out motifs with multiple-testing corrected p-values > 0.1.

PeaKO computes a ranking metric *r* that represents the proportion of high-quality $A_{\text{WT}}$ peaks found in set *B* but not in set $A_{\text{KO}}$. To do this, peaKO calculates the overlap between peak sets $A_{\text{WT}}$ and $A_{\text{KO}}$ from Pipeline A and peak set *B* from Pipeline B through a series of set operations:


$$r=\frac{\left|(A_{\text{WT}}-A_{\text{KO}})\cap B\right|}{\left|A_{\text{WT}}\right|}.$$

PeaKO performs this by employing pybedtools (version 0.7.7; BEDTools version 2.26.0) (Quinlan and Hall, 2010; Dale *et al.*, 2011). First, peaKO removes any $A_{\text{WT}}$ peak overlapping at least 1 bp of an $A_{\text{KO}}$ peak (pybedtoolssubtract -A; Fig. 1B). Second, peaKO finds regions overlapping by at least 1 bp between remaining $A_{\text{WT}}$ peaks and *B* peaks (pybedtoolsintersect -wa). Third, peaKO applies pybedtoolsmerge with default settings to overlapping regions, which merges identical regions and ensures that the ranking metric *r* has a maximum value of 1. PeaKO’s final output consists of a list of motifs ranked according to this metric.

### 5.3 Datasets

We analyzed a total of eight publicly available ChIP-seq experiment datasets with KO controls (Table 1). Of these eight, we selected two datasets (GATA3 and SRF) from Krebs *et al.* (2014), while we selected the remainder by searching for KO-associated ChIP-seq datasets on Gene Expression Omnibus (GEO) (Edgar *et al.*, 2002). We accessed datasets through GEO, except for the SRF dataset (Heinz *et al.*, 2010), available on Zenodo (https://doi.org/10.5281/zenodo.3405482). ATF3 experiments come from human tissue, while the other experiments come from mouse tissue.

### 5.4 Motifs

We downloaded the collection of vertebrate motifs in MEME format (Bailey *et al.*, 2015) from the JASPAR CORE 2016 motif database, which consists of curated PWMs derived from *in vivo* and *in vitro* methods (Mathelier *et al.*, 2016).

We defined each canonical motif from the JASPAR collection as the motif matching the target transcription factor except in two cases: OCT4 and CHOP (Table 2). In both cases, we instead chose motifs derived from their common heterodimer complex forms. CHOP or DDIT3 likely binds DNA as an obligate multimer (Newman and Keating, 2003; Lambert *et al.*, 2018), so we used Ddit3::Cebpa (MA0019.1). The CHOP monomer motif closely resembles its C/EBP*α* heterodimer motif, relative to its Cis-BP (version 1.02) (Weirauch *et al.*, 2014) DDIT3 motif [T025314_1.02, derived from HOCOMOCO (Kulakovskiy *et al.*, 2013b)]. For OCT4, we used the Pou5f1::Sox2 motif (MA0142.1; see Discussion). We confirmed that none of the target motifs derived from ChIP-seq datasets included in our analyses. To avoid redundancies from Cis-BP and HOCOMOCO motif databases, we decided to use only JASPAR motifs.

We provided motifs to CentriMo (Bailey and Machanick, 2012; Lesluyes *et al.*, 2014) for central enrichment analyses and to Tomtom (Gupta *et al.*, 2007) for similarity assessments.

### 5.5 Pre-processing, alignment and peak calling

Before alignment, we trimmed adapter sequences with Trim Galore (version 0.4.1) (Krueger, 2012) which uses Cutadapt (version 1.8.3) (Martin, 2011). We assessed sequencing data quality using FastQC (version 0.11.5) (Andrews, 2018). We used Picard’s FixMateInformation and AddOrReplaceReadsGroups (version 2.10.5) (Broad Institute, 2015) and GATK’s PrintReads (version 3.6) (McKenna *et al.*, 2010) to prevent GATK errors. Then, we aligned reads to GRCm38/mm10 (Church *et al.*, 2011) or GRCh38/hg38 (Schneider *et al.*, 2017) with BWA bwa-aln (version 0.7.15) (Li and Durbin, 2009), as recommended (Li and Durbin, 2019), since some datasets have reads ≪ 70 bp. We used Sambamba (version 0.6.6) (Tarasov *et al.*, 2015) for post-processing.

Next, we called peaks using MACS2 (version 2.0.10) (Zhang *et al.*, 2008) with parameters -q 0.05. In Pipeline A, we called WT and KO peaks separately. In Pipeline B, we provided the KO dataset as a control to the WT dataset during peak calling (parameter -c), resulting in a single set of peaks. We kept all peaks in each set and performed no additional filtering.

### 5.6 Combining replicates

For MEF2D, OCT4, and TEAD4 experiments which consist of biological replicates (see Table 1), we processed replicates using the ENCODE Transcription Factor and Histone ChIP-seq processing pipeline (Kundaje *et al.*, 2018). The ENCODE pipeline replaces the pre-processing, alignment, and peak calling steps described earlier. We chose default parameters for punctate (narrow peak) binding experiments in all steps. Instead of a q-value threshold, this pipeline caps the number of peaks (n=500 000) to ensure that the IDR framework (Li *et al.*, 2011) can analyze a sufficient number of peaks across a full spectrum. IDR combines peaks across replicates based on the assumption that strong peaks shared across replicates represent true binding events, while weak, one-off peaks represent noise. To emulate the first steps of Pipeline A and Pipeline B, we either ran the ENCODE pipeline on WT replicates and KO replicates separately (for Pipeline A), or we ran the ENCODE pipeline on all WT and KO replicates simultaneously, setting KO replicates as controls (for Pipeline B). For downstream motif analyses, we used the combined ‘optimal’ peak sets output by IDR.

### 5.7 Motif analyses with MEME-ChIP

In both pipelines, we employed MEME-ChIP (Ma *et al.*, 2014; Machanick and Bailey, 2011) from the MEME Suite (Bailey *et al.*, 2009, 2015) for motif analysis. We used MEME-ChIP version 4.12.0, except for CentriMo, which we compiled from version 4.11.2 and modified to output negative sequences. MEME-ChIP performs motif discovery with complementary algorithms MEME (Bailey and Elkan, 1994, 1995) and DREME (Bailey, 2011), and motif enrichment with CentriMo (Bailey and Machanick, 2012; Lesluyes *et al.*, 2014).

We extended MACS2 narrowPeak regions equidistantly from peak summits to create a uniform set of 500 bp centered peaks (Ma *et al.*, 2014). Then, we extracted underlying genomic sequences using BEDTools slop (version 2.23.0) (Quinlan and Hall, 2010) from a repeat-masked genome. We masked the genome with Tandem Repeats Finder (version 4.09) (Benson, 1999) with options -hm-ngs and parameters 2 7 7 80 10 50 500 for mouse [as done originally by Benson (1999)], and parameters 2 5 5 80 10 30 200 for human [as recommended by Frith *et al.* (2010)].

In Pipeline A, we provided the negative control set in addition to the WT set, running MEME, DREME, and CentriMo in differential mode. In ranking known motifs, we ran CentriMo providing only JASPAR database motifs. Differential CentriMo mode ranks motifs according to Fisher E-values. Since the E-value is the p-value (at most 1) times the number of tests, the E-value cannot exceed the number of motifs in the provided database. Once differential CentriMo reaches the maximum E-value, it starts ranking motifs alphanumerically by motif identifier. Therefore, we do not consider the reported, but relatively meaningless, ranks of motifs with non-significant Fisher E-values. In ranking *de novo* motifs, we ran CentriMo providing only MEME and DREME motifs.

### 5.8 Pooling *de novo* motifs

Each run of MEME or DREME creates new and globally non-unique identifiers for output motifs. This leads to recurring identifiers that refer to different motifs across multiple runs. To consolidate identifiers across multiple MEME and DREME runs, we modified identifiers to reflect the pipeline from which they originate. We then pooled *de novo* motifs across methods and re-ran the CentriMo step of each pipeline, providing the pooled database, allowing for accurate comparisons.

### 5.9 Assessing similarity of *de novo* motifs to known motifs

For each experiment, we quantified the similarity of *de novo* motifs to the known JASPAR motif using Tomtom (Gupta *et al.*, 2007). Tomtom compared the *de novo* motifs with the JASPAR motif database through ungapped alignment across columns (Gupta *et al.*, 2007). Tomtom generated a list of known motif matches, ranked by increasing Bonferroni-corrected p-values. An exact match between a *de novo* motif and a JASPAR motif would result in the JASPAR motif’s ranking first in this list of matches.

### 5.10 Comparing input to KO controls

For experiments with associated input controls, we re-ran our known motif and *de novo* motif analyses swapping out KO datasets for input datasets. We compared peaks between sets using UpSet (version 1.4.0) plots (Lex *et al.*, 2014), via Intervene (version 0.6.2) (Khan and Mathelier, 2017), which calculates genomic region overlaps with BEDTools (version 2.26.0) (Quinlan and Hall, 2010).

## Data Availability

PeaKO is available at https://peako.hoffmanlab.org. We additionally include Python source code for peaKO and both pipelines at: https://github.com/hoffmangroup/peako. Persistent availability is ensured by Zenodo, in which we have deposited the version of our code we used (https://doi.org/10.5281/zenodo.3338324), its downstream CentriMo and peaKO outputs (https://doi.org/10.5281/zenodo.3338330), and our changes to the CentriMo source code and the Linux x86-64 binary that we used (https://doi.org/10.5281/zenodo.3356995). All source code is licensed under a GNU General Public License, version 3 (GPLv3), except for CentriMo, which retains its original license.

## References

[vbad031-B1] Aksoy I. et al (2013) Oct4 switches partnering from Sox2 to Sox17 to reinterpret the enhancer code and specify endoderm. EMBO J., 32, 938–953. doi:10.1038/emboj.2013.31 23474895 PMC3616284

[vbad031-B2] Andrews S. (2018) FastQC: a quality control tool for high throughput sequence data. https://www.bioinformatics.babraham.ac.uk/projects/fastqc

[vbad031-B3] Andzelm M.M. et al (2015) MEF2D drives photoreceptor development through a genome-wide competition for tissue-specific enhancers. Neuron, 86, 247–263. doi:10.1016/j.neuron.2015.02.038 25801704 PMC4393375

[vbad031-B4] Auerbach R.K. et al (2009) Mapping accessible chromatin regions using Sono-Seq. Proc. Natl. Acad. Sci. USA, 106, 14926–14931. doi:10.1073/pnas.0905443106 19706456 PMC2736440

[vbad031-B5] Bailey T. et al (2013) Practical guidelines for the comprehensive analysis of ChIP-seq data. PLoS Comput. Biol., 9, e1003326. doi:10.1371/journal.pcbi.1003326 24244136 PMC3828144

[vbad031-B6] Bailey T.L. (2011) DREME: motif discovery in transcription factor ChIP-seq data. Bioinformatics, 27, 1653–1659. doi:10.1093/bioinformatics/btr261 21543442 PMC3106199

[vbad031-B7] Bailey T.L. , Elkan C. (1994) Fitting a mixture model by expectation maximization to discover motifs in biopolymers. *Proc. Int. Conf. Intell. Syst. Mol. Biol.* 2, 28–36. 7584402

[vbad031-B8] Bailey T.L. , Elkan C. (1995) Unsupervised learning of multiple motifs in biopolymers using expectation maximization. Mach. Learn. 21, 51–80. doi:10.1007/BF00993379

[vbad031-B9] Bailey T.L. , Machanick P. (2012) Inferring direct DNA binding from ChIP-seq. Nucleic Acids Res., 40, e128. doi:10.1093/nar/gks433 22610855 PMC3458523

[vbad031-B10] Bailey,T.L. et al (2009) MEME Suite: tools for motif discovery and searching. Nucleic Acids Res. 37, W202–W208. doi:10.1093/nar/gkp335 19458158 PMC2703892

[vbad031-B11] Bailey,T.L. et al (2015) The MEME Suite. Nucleic Acids Res. 43, W39–W49. doi:10.1093/nar/gkv416 25953851 PMC4489269

[vbad031-B12] Benos P.V. et al (2002) Additivity in protein–DNA interactions: how good an approximation is it? Nucleic Acids Res., 30, 4442–4451. doi:10.1093/nar/gkf578 12384591 PMC137142

[vbad031-B13] Benson G. (1999) Tandem repeats finder: a program to analyze DNA sequences. Nucleic Acids Res., 27, 573–580. doi:10.1093/nar/27.2.573 9862982 PMC148217

[vbad031-B14] Berg O.G. , von Hippel P.H. (1987) Selection of DNA binding sites by regulatory proteins. J. Mol. Biol., 193, 723–750. doi:10.1016/0022-2836(87)90354-8 3612791

[vbad031-B15] Broad Institute. (2015) Picard. https://broadinstitute.github.io/picard

[vbad031-B16] Chen L. et al (2015) A novel statistical method for quantitative comparison of multiple ChIP-seq datasets. Bioinformatics, 31, 1889–1896. doi:10.1093/bioinformatics/btv094 25682068 PMC4542775

[vbad031-B17] Chen Y. et al (2012) Systematic evaluation of factors influencing ChIP-seq fidelity. Nat. Methods, 9, 609–614. doi:10.1038/nmeth.1985 22522655 PMC3477507

[vbad031-B78613382] Church,D.M. et al (2011) Modernizing reference genome assemblies. PLoS Biol. 9, e1001091. doi:10.1371/journal.pbio.1001091 21750661 PMC3130012

[vbad031-B18] Cong,L. et al (2013) Multiplex genome engineering using CRISPR/Cas systems. Science 339, 819–823. doi:10.1126/science.1231143 23287718 PMC3795411

[vbad031-B19] Dale,R.K. et al (2011) Pybedtools: A flexible Python library for manipulating genomic datasets and annotations. Bioinformatics 27, 3423–3424. doi:10.1093/bioinformatics/btr539 21949271 PMC3232365

[vbad031-B20] Doudna J.A. , Charpentier E. (2014) The new frontier of genome engineering with CRISPR-Cas9. Science, 346, 1258096. doi:10.1126/science.1258096 25430774

[vbad031-B21] Dror I. et al (2016) How motif environment influences transcription factor search dynamics: finding a needle in a haystack. Bioessays, 38, 605–612. doi:10.1002/bies.201600005 27192961 PMC5023137

[vbad031-B22] Edgar R. et al (2002) Gene expression omnibus: NCBI gene expression and hybridization array data repository. Nucleic Acids Res., 30, 207–210. doi:10.1093/nar/30.1.207 11752295 PMC99122

[vbad031-B23] Eder T. , Grebien F. (2022) Comprehensive assessment of differential ChIP-seq tools guides optimal algorithm selection. Genome Biol., 23, 119. doi:10.1186/s13059-022-02686-y 35606795 PMC9128273

[vbad031-B24] ENCODE Project Consortium. (2012) An integrated encyclopedia of DNA elements in the human genome. Nature, 489, 57–74. doi:10.1038/nature11247 22955616 PMC3439153

[vbad031-B25] Fornes O. et al (2020) JASPAR 2020: update of the open-access database of transcription factor binding profiles. Nucleic Acids Res., 48, D87–D92. doi:10.1093/nar/gkz1001 31701148 PMC7145627

[vbad031-B26] Frith M.C. et al (2010) Parameters for accurate genome alignment. BMC Bioinformatics, 11, 80. doi:10.1186/1471-2105-11-80 20144198 PMC2829014

[vbad031-B27] Furey T.S. (2012) ChIP-seq and beyond: new and improved methodologies to detect and characterize protein–DNA interactions. Nat. Rev. Genet., 13, 840–852. doi:10.1038/nrg3306 23090257 PMC3591838

[vbad031-B28] Gupta S. et al (2007) Quantifying similarity between motifs. Genome Biol., 8, R24. doi:10.1186/gb-2007-8-2-r24 17324271 PMC1852410

[vbad031-B29] Han J. et al (2013) ER-stress-induced transcriptional regulation increases protein synthesis leading to cell death. Nat. Cell Biol., 15, 481–490. doi:10.1038/ncb2738 23624402 PMC3692270

[vbad031-B30] Head S.R. et al (2014) Library construction for next-generation sequencing: overviews and challenges. Biotechniques, 56, 61–77. doi:10.2144/000114133 24502796 PMC4351865

[vbad031-B31] Heinz S. et al (2010) Simple combinations of lineage-determining transcription factors prime *cis*-regulatory elements required for macrophage and B cell identities. Mol. Cell., 38, 576–589. doi:10.1016/j.molcel.2010.05.004 20513432 PMC2898526

[vbad031-B32] Johnson D.S. et al (2007) Genome-wide mapping of *in vivo* protein–DNA interactions. Science, 316, 1497–1502. doi:10.1126/science.1141319 17540862

[vbad031-B34] Joshi S. et al (2017) TEAD transcription factors are required for normal primary myoblast differentiation *in vitro* and muscle regeneration *in vivo*. PLoS Genet., 13, e1006600. doi:10.1371/journal.pgen.1006600 28178271 PMC5323021

[vbad031-B35] Khan A. , Mathelier A. (2017) Intervene: a tool for intersection and visualization of multiple gene or genomic region sets. BMC Bioinformatics, 18, 287. doi:10.1186/s12859-017-1708-7 28569135 PMC5452382

[vbad031-B36] Kidder B.L. et al (2011) ChIP-Seq: technical considerations for obtaining high-quality data. Nat. Immunol., 12, 918–922. doi:10.1038/ni.2117 21934668 PMC3541830

[vbad031-B37] King H.W. , Klose R.J. (2017) The pioneer factor OCT4 requires the chromatin remodeller BRG1 to support gene regulatory element function in mouse embryonic stem cells. eLife, 6, e22631. doi:10.7554/eLife.22631 28287392 PMC5400504

[vbad031-B38] Krebs W. et al (2014) Optimization of transcription factor binding map accuracy utilizing knockout-mouse models. Nucleic Acids Res., 42, 13051–13060. doi:10.1093/nar/gku1078 25378309 PMC4245947

[vbad031-B39] Krueger F. (2012) Trim Galore! https://www.bioinformatics.babraham.ac.uk/projects/trim_galore/

[vbad031-B40] Kulakovskiy I. et al (2013a) From binding motifs in ChIP-Seq data to improved models of transcription factor binding sites. J. Bioinform. Comput. Biol., 11, 1340004. doi:10.1142/S0219720013400040 23427986

[vbad031-B41] Kulakovskiy I.V. et al (2013b) HOCOMOCO: a comprehensive collection of human transcription factor binding sites models. Nucleic Acids Res., 41, D195–D202. doi:10.1093/nar/gks1089 23175603 PMC3531053

[vbad031-B42] Kundaje A. et al (2018) ENCODE transcription factor and histone ChIP-Seq processing pipeline. https://github.com/ENCODE-DCC/chip-seq-pipeline2.

[vbad031-B43] Lai,E. et al (1993) Hepatocyte nuclear factor 3/fork head or ‘winged helix’ proteins: a family of transcription factors of diverse biologic function. Proc. Natl. Acad. Sci. U S A 90, 10421–10423. doi:10.1073/pnas.90.22.10421 8248124 PMC47788

[vbad031-B44] Lambert S.A. et al (2018) The human transcription factors. Cell, 172, 650–665. doi:10.1016/j.cell.2018.01.029 29425488 PMC12908702

[vbad031-B45] Lambert S.A. et al (2019) Similarity regression predicts evolution of transcription factor sequence specificity. Nat. Genet., 51, 981–989. doi:10.1038/s41588-019-0411-1 31133749

[vbad031-B46] Landt S.G. et al (2012) ChIP-seq guidelines and practices of the ENCODE and modENCODE consortia. Genome Res., 22, 1813–1831. doi:10.1101/gr.136184.111 22955991 PMC3431496

[vbad031-B47] Lesluyes T. et al (2014) Differential motif enrichment analysis of paired ChIP-seq experiments. BMC Genomics, 15, 752. doi:10.1186/1471-2164-15-752 25179504 PMC4167127

[vbad031-B48] Lex A. et al (2014) UpSet: visualization of intersecting sets. IEEE Trans. Vis. Comput. Graph., 20, 1983–1992. doi:10.1109/TVCG.2014.2346248 26356912 PMC4720993

[vbad031-B49] Li H. , Durbin R. (2009) Fast and accurate short read alignment with Burrows–Wheeler transform. Bioinformatics, 25, 1754–1760. doi:10.1093/bioinformatics/btp324 19451168 PMC2705234

[vbad031-B50] Li H. , Durbin R. (2019) BWA. https://github.com/lh3/bwa

[vbad031-B51] Li Q. et al (2011) Measuring reproducibility of high-throughput experiments. Ann. Appl. Stat., 5, 1752–1779. doi:10.1214/11-AOAS466

[vbad031-B52] Love M.I. et al (2014) Moderated estimation of fold change and dispersion for RNA-seq data with DESeq2. Genome Biol., 15, 550. doi:10.1186/s13059-014-0550-8 25516281 PMC4302049

[vbad031-B53] Lun,A.T.L and Smyth,G.K. (2016) csaw: A Bioconductor package for differential binding analysis of chip-seq data using sliding windows. Nucleic Acids Res. 44, e45. doi:10.1093/nar/gkv1191 26578583 PMC4797262

[vbad031-B54] Ma W. et al (2014) Motif-based analysis of large nucleotide data sets using MEME-ChIP. Nat. Protoc., 9, 1428–1450. doi:10.1038/nprot.2014.083 24853928 PMC4175909

[vbad031-B55] Machanick P. , Bailey T.L. (2011) MEME-ChIP: motif analysis of large DNA datasets. Bioinformatics, 27, 1696–1697. doi:10.1093/bioinformatics/btr189 21486936 PMC3106185

[vbad031-B56] Martin M. (2011) Cutadapt removes adapter sequences from high-throughput sequencing reads. EMBnet J., 17, 10–12. doi:10.14806/ej.17.1.200

[vbad031-B57] Mathelier A. et al (2016) JASPAR 2016: a major expansion and update of the open-access database of transcription factor binding profiles. Nucleic Acids Res., 44, D110–D115. doi:10.1093/nar/gkv1176 26531826 PMC4702842

[vbad031-B58] McKenna A. et al (2010) The Genome Analysis Toolkit: a MapReduce framework for analyzing next-generation DNA sequencing data. Genome Res., 20, 1297–1303. doi:10.1101/gr.107524.110 20644199 PMC2928508

[vbad031-B59] Merika M. , Orkin S.H. (1993) DNA-binding specificity of GATA family transcription factors. Mol. Cell. Biol., 13, 3999–4010. doi:10.1128/mcb.13.7.3999 8321207 PMC359949

[vbad031-B60] Mitchell P.J. , Tjian R. (1989) Transcriptional regulation in mammalian cells by sequence-specific DNA binding proteins. Science, 245, 371–378. doi:10.1126/science.2667136 2667136

[vbad031-B61] Newman J.R.S. , Keating A.E. (2003) Comprehensive identification of human bZIP interactions with coiled-coil arrays. Science, 300, 2097–2101. doi:10.1126/science.1084648 12805554

[vbad031-B62] Park P.J. (2009) ChIP-seq: advantages and challenges of a maturing technology. Nat. Rev. Genet., 10, 669–680. doi:10.1038/nrg2641 19736561 PMC3191340

[vbad031-B63] Pepke S. et al (2009) Computation for ChIP-seq and RNA-seq studies. Nat. Methods, 6, S22–S32. doi:10.1038/nmeth.1371 19844228 PMC4121056

[vbad031-B64] Quinlan A.R. , Hall I.M. (2010) BEDTools: a flexible suite of utilities for comparing genomic features. Bioinformatics, 26, 841–842. doi:10.1093/bioinformatics/btq033 20110278 PMC2832824

[vbad031-B65] Rastogi C. et al (2018) Accurate and sensitive quantification of protein–DNA binding affinity. Proc. Natl. Acad. Sci. USA, 115, E3692–E3701. doi:10.1073/pnas.1714376115 29610332 PMC5910815

[vbad031-B66] Robertson G. et al (2007) Genome-wide profiles of STAT1 DNA association using chromatin immunoprecipitation and massively parallel sequencing. Nat. Methods, 4, 651–657. doi:10.1038/nmeth1068 17558387

[vbad031-B67] Robinson M.D. et al (2010) edgeR: a Bioconductor package for differential expression analysis of digital gene expression data. Bioinformatics, 26, 139–140. doi:10.1093/bioinformatics/btp616 19910308 PMC2796818

[vbad031-B3574644] Rodríguez-Martínez,J.A. et al (2017) Combinatorial bZIP dimers display complex DNA-binding specificity landscapes. eLife 6, e19272. doi:10.7554/eLife.19272 PMC5349851 28186491

[vbad031-B68] Sandelin A. , Wasserman W.W. (2004) Constrained binding site diversity within families of transcription factors enhances pattern discovery bioinformatics. J. Mol. Biol., 338, 207–215. doi:10.1016/j.jmb.2004.02.048 15066426

[vbad031-B69] Savic D. et al (2015) CETCh-seq: CRISPR epitope tagging ChIP-seq of DNA-binding proteins. Genome Res., 25, 1581–1589. doi:10.1101/gr.193540.115 26355004 PMC4579343

[vbad031-B163269] Schneider,V.A. et al (2017) Evaluation of GRCh38 and de novo haploid genome assemblies demonstrates the enduring quality of the reference assembly. Genome Res. 27, 849–864. doi:10.1101/gr.213611.116 28396521 PMC5411779

[vbad031-B70] Schneider T.D. , Stephens R.M. (1990) Sequence logos: a new way to display consensus sequences. Nucleic Acids Res., 18, 6097–6100. doi:10.1093/nar/18.20.6097 2172928 PMC332411

[vbad031-B71] Schwenk F. et al (1995) A *cre*-transgenic mouse strain for the ubiquitous deletion of *loxP*-flanked gene segments including deletion in germ cells. Nucleic Acids Res., 23, 5080–5081. doi:10.1093/nar/23.24.5080 8559668 PMC307516

[vbad031-B72] Skene P.J. et al (2018) Targeted *in situ* genome-wide profiling with high efficiency for low cell numbers. Nat. Protoc., 13, 1006–1019. doi:10.1038/nprot.2018.015 29651053

[vbad031-B73] Stark R. , Brown G. (2011) DiffBind: differential binding analysis of ChIP-Seq peak data. https://bioconductor.org/packages/release/bioc/vignettes/DiffBind/inst/doc/DiffBind.pdf

[vbad031-B74] Sternberg N. , Hamilton D. (1981) Bacteriophage P1 site-specific recombination. J. Mol. Biol., 150, 467–486. doi:10.1016/0022-2836(81)90375-2 6276557

[vbad031-B75] Sullivan A.L. et al (2011) Serum response factor utilizes distinct promoter- and enhancer-based mechanisms to regulate cytoskeletal gene expression in macrophages. Mol. Cell. Biol., 31, 861–875. doi:10.1128/MCB.00836-10 21135125 PMC3028656

[vbad031-B76] Tarasov A. et al (2015) Sambamba: fast processing of NGS alignment formats. Bioinformatics, 31, 2032–2034. doi:10.1093/bioinformatics/btv098 25697820 PMC4765878

[vbad031-B77] Tu S. , Shao Z. (2017) An introduction to computational tools for differential binding analysis with ChIP-seq data. Quant. Biol., 5, 226–235. doi:10.1007/s40484-017-0111-8

[vbad031-B78] Viger R.S. et al (2008) Role of the GATA family of transcription factors in endocrine development, function, and disease. Mol. Endocrinol., 22, 781–798. doi:10.1210/me.2007-0513 18174356 PMC2276466

[vbad031-B79] Wei G. et al (2011) Genome-wide analyses of transcription factor GATA3-mediated gene regulation in distinct T cell types. Immunity, 35, 299–311. doi:10.1016/j.immuni.2011.08.007 21867929 PMC3169184

[vbad031-B80] Weirauch M.T. et al (2014) Determination and inference of eukaryotic transcription factor sequence specificity. Cell, 158, 1431–1443. doi:10.1016/j.cell.2014.08.009 25215497 PMC4163041

[vbad031-B81] Worsley Hunt R. , Wasserman W.W. (2014) Non-targeted transcription factors motifs are a systemic component of ChIP-seq datasets. Genome Biol., 15, 412. doi:10.1186/s13059-014-0412-4 25070602 PMC4165360

[vbad031-B82] Zeineddine D. et al (2014) The Oct4 protein: more than a magic stemness marker. Am. J. Stem Cells, 3, 74–82. 25232507 PMC4163606

[vbad031-B83] Zhang Y. et al (2014) PePr: a peak-calling prioritization pipeline to identify consistent or differential peaks from replicated ChIP-seq data. Bioinformatics, 30, 2568–2575. doi:10.1093/bioinformatics/btu372 24894502 PMC4155259

[vbad031-B84] Zhang Y. et al (2008) Model-based analysis of ChIP-Seq (MACS). Genome Biol., 9, R137. doi:10.1186/gb-2008-9-9-r137 18798982 PMC2592715

[vbad031-B85] Zhao J. et al (2016) The common stress responsive transcription factor ATF3 binds genomic sites enriched with p300 and H3K27ac for transcriptional regulation. BMC Genomics, 17, 335. doi:10.1186/s12864-016-2664-8 27146783 PMC4857411

